# Domino Flap Using Bilobed Flap for Large Scapular Flap Donor Site: A Case Report

**DOI:** 10.1055/a-2640-4061

**Published:** 2025-08-29

**Authors:** Meirizal Meirizal, Ardicho Irfantian, A. Faiz Huwaidi, Agung Susilo Lo

**Affiliations:** 1 Department of Orthopedics and Traumatology, Rumah Sakit Umum Pemerintah Dr. Sardjito Hospital, Sleman, D.I. Yogyakarta, Indonesia; 2 Faculty of Medicine, Public Health and Nursing, Universitas Gadjah Mada, Jl. Farmako, Sendowo, Sekip Utara, Sleman, D.I. Yogyakarta, Indonesia

**Keywords:** bilobed flap, scapular flap, reconstruction

## Abstract

Scapular flaps are favored for large soft tissue defects, but with a high risk of shoulder contracture. This case report presents a novel application of a bilobed flap technique to cover the donor site after harvesting a 23-cm scapular flap. The patient was a 25-year-old male with a right-hand open degloving injury measuring 25 × 22 cm. The scapular flap was planned to cover the soft tissue defect, and the bilobed flap was used to close the donor site. After 6 months, the left shoulder showed full range of motion, and the scapular flap was viable with good right-hand function. This case introduces the largest bilobed flap used to date. The report emphasizes two main advantages, it prevents shoulder contracture and allows surgeons to exceed the traditionally recommended flap size with minimal donor site morbidity. The large bilobed flap has proven effective in closing donor site defects following scapular flap harvest, demonstrating satisfactory outcomes. Additionally, this technique can be utilized to address other donor site defects.

## Introduction

Traumatic wounds require effective management to prevent limb-threatening conditions and potential amputation. Adequate soft tissue coverage not only restores limb function but also improves aesthetic appearance. Free flaps are the most versatile option for covering large soft tissue defects, including latissimus dorsi, anterolateral thigh (ALT), scapular, and parascapular flaps. 1

The scapular flap offers several advantages over other flaps, but harvesting a larger scapular flap than recommended poses challenges due to donor site morbidity. 2 3 For large defects, primary closure becomes difficult. 4 Skin grafts alone are inadequate as they often result in seroma formation at the donor site. 2 The bilobed flap, first introduced by Dr. J.F.S. Esser in 1918, was initially used for small soft tissue defects, such as basal cell carcinoma on the nose. Our recent study successfully modified the bilobed flap to close larger soft tissue defects in amputated stumps. 5

The restoration of a thin, pliable soft tissue envelope is essential for reestablishing mobility, achieving optimal functional outcomes, and preventing infection. 1 Originally used for nasal reconstruction, the bilobed flap has been applied to other defects, but it has not yet restored patients' range of motion. 6 7 This case report highlights the effectiveness of large scapular flaps in addressing soft tissue defects in the hand, as well as the efficacy of bilobed flaps in minimizing donor site morbidity.

## Case

A 25-year-old male student presented to our emergency room following a traffic accident. The patient was right-handed and had no known comorbidities. He arrived with stable vital signs and exhibited an open skin degloving injury on his right hand, accompanied by a total rupture of the second to fifth extensor digitorum muscles. His distal oxygen saturation was 98%.

The patient consented to undergo the operative procedure. Our primary objective was to close the soft tissue defect on the patient's right hand, which measured approximately 25 × 33 cm after the debridement procedure, exposing muscle and subcutaneous tissue. With a wound bed with a muscular base which still viable after debridement, we utilized a scapular flap to close the site defect. Large defects can be seen on both sides, dorsal and palmar, so not only the scapular flap but also the oblique parascapular flap was obtained. The maximum donor size that did not cause contracture and limited range of motion in shoulder abduction is 20 cm. Large donor with double flap (scapular and parascapular) with vertical incision, resulting in stitches that are too tight, even worse, the donor cannot be closed primarily, and of course, it will have the effect of disrupting shoulder abduction. So, with this problem, we designed and applied a bilobed flap for donor closure. Due to the extensive defect on the hand and the need for thin tissue to facilitate fine motor movements, we chose the scapular flap over other flap options. This choice was based on the scapular flap's ability to provide the necessary tissue characteristics for both functional restoration and aesthetic preservation of the hand.

**Fig. 1:**
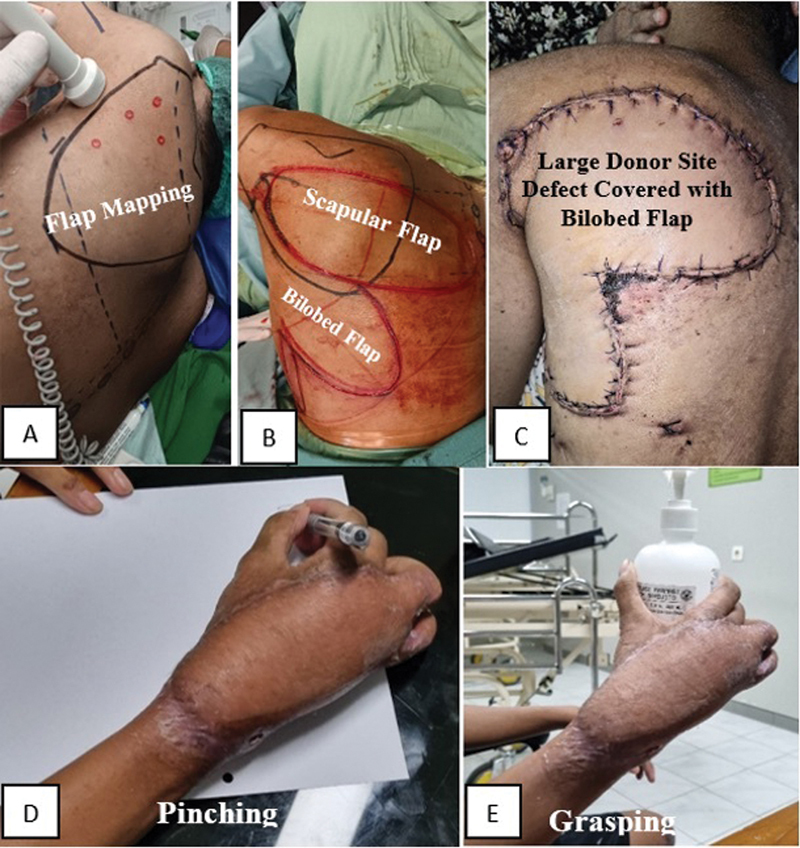
The figure demonstrates the reconstruction procedure and clinical function after a 6-month follow-up ( **A** - **E** ).

The patient was positioned in the lateral decubitus position with the left shoulder superior. The right arm was positioned with 90 degrees of abduction and full elbow extension. We mapped the scapular flap with a design, using Doppler to identify the associated pedicle, the dorsal circumflex scapular artery ( Fig. 1A, B ). Since the harvested scapular flap exceeded 9 cm, primary closure was not feasible. We used a bilobed flap to close the donor site defect. The mapping and design of the bilobed flap were completed before harvesting the scapular flap to match the defect size ( Fig. 1B, C ).

**Fig. 2:**
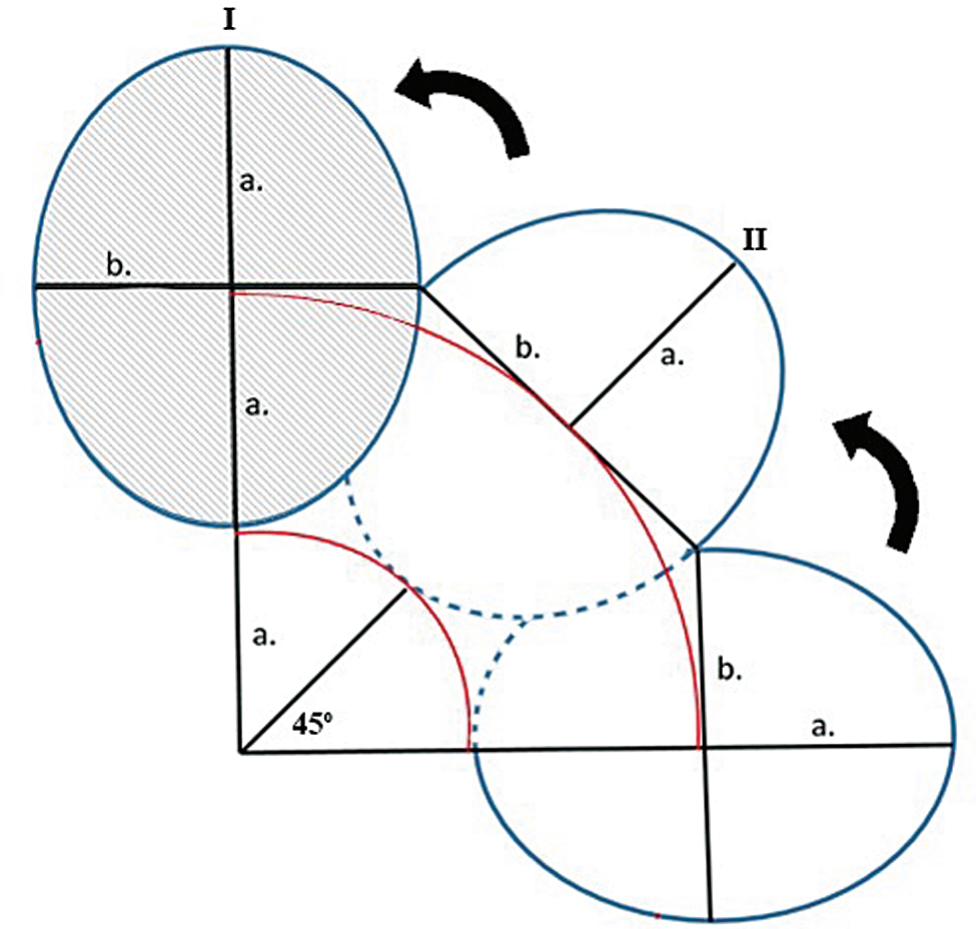
Schematic figure of bilobed flap.

The first lobe (II) closes the original hand defect (I), while the second lobe (III) covers the first lobe defect, with its own defect closed primarily In a bilobed flap, both lobes have a height of 2a and a width of 2b, with the first lobe oriented at 45 degrees and the second at 90 degrees, both pointing outward from the fulcrum. These proportions and angles ensure optimal rotation, defect coverage, minimal tension, and preservation of blood flow ( Fig. 2 ).

**Fig. 3:**
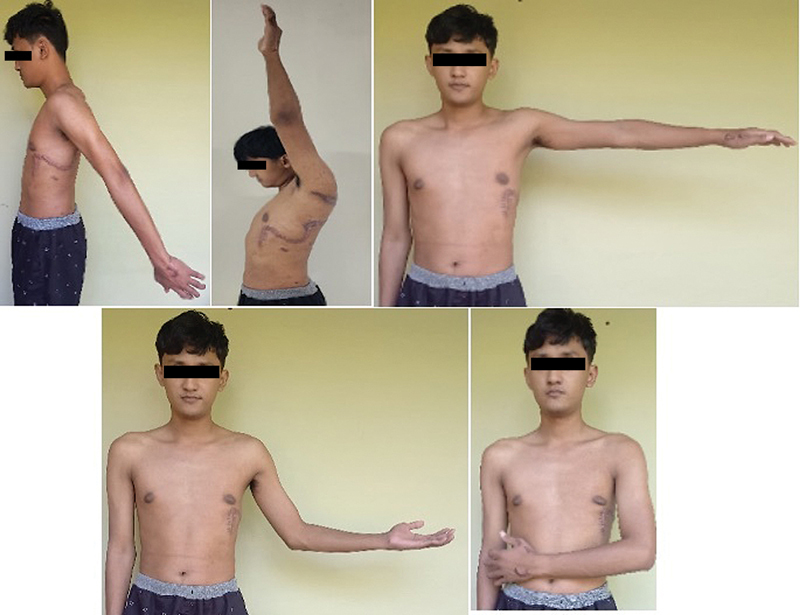
Six-month postoperative shoulder range of motion assessment showing full functionality in forward elevation, extension, abduction, external, and internal rotation.

Following the surgery, a drain was placed to prevent seroma accumulation. On average, the drainage was 200 mL per day, gradually tapering off after 3 days. There was no donor site morbidity and the flap was viable. We conducted follow-up and at 6 months, the patient showed good clinical progress with a viable scapular flap and excellent hand function ( Fig. 1 ( D ) and ( E )), reflected by a DASH score of 9.1/100. No donor site complications, such as seroma or flap necrosis, were observed, and the patient's left shoulder had normal function with full range of motion. To date, the patient continues to undergo regular follow-up evaluations ( Fig. 3 and Table 1 ). Written informed consent was obtained from the patient for publication of this case report and accompanying images.

**Table 1:** Table showing the outcome after a 6-month follow-up

Parameter	Preoperative	Postoperative
ROM of shoulder joint		
Forward flexion	180	180
Extension	60	60
Internal rotation	70	80
External rotation	90	90
Abduction	180	180
Flap viability	–	Viable
Keloid/hypertrophic scar	–	++
Infection	–	−
Quick-Dash score	6.8	9.1

Abbreviation: ROM, range of motion.

## Discussion

Flaps vascularized by the subscapular artery system are widely used to cover large soft tissue defects. The scapular flap was first published in the 1980s and vascularized by the subscapular artery system and has two main branches: the circumflex scapular artery and the thoracodorsal artery. The circumflex scapular artery supplies blood to the parascapular fasciocutaneous flap and the lateral part of the scapular bone. The distal portion of the scapula is vascularized by the angular branch of the thoracodorsal artery. 6 7 This versatility led us to choose the subscapular flap system for closing the patient's soft tissue defect. Anatomically, we can harvest a scapular flap up to 20 cm in length, which is large for this type of flap. Moreover, the scapular flap preserves shoulder joint stability, strength, and range of motion, unlike the latissimus dorsi muscular or musculocutaneous flap. The scapular flap is thin, flexible, and durable, providing a more aesthetically pleasing outcome compared to the ALT-free flap, which often requires debulking. 2 3

The important issue encountered with harvesting large flaps is managing donor site defects. Common methods such as split-thickness skin grafting and full-thickness skin grafting are often used to close these defects, but they can lead to wound complications and aesthetic issues. 8 9 The chimeric flap technique offers a solution for defects when propeller flap technique is not suitable, defect resection that requires an amount of skin paddle, and defects that benefit from dual skin paddles (external and intraoral defect). 10 However, seroma formation has been reported in defects resulting from harvesting chimeric scapular flaps for maxilla reconstruction. 11 Additionally, direct suturing is not feasible for defects larger than 9 cm. 4 To address these challenges, we employed a bilobed flap for the primary closure of donor site morbidity in scapular flap defects. In a large defect of amputation stump coverage, bilobed flap results in low site donor morbidity with well-padded prosthesis fitting. The bilobed flap design is used to cover a small defect using the epidermis, dermis, and subcutaneous tissue, but for the flap to be able to cover a large area and secure blood supply, fascia needs to be included. 5

The bilobed flap was first described in 1918 by Esser, using a rotation angle of up to 180 degrees. It was later modified by reducing the rotation angle to 90 to 110 degrees to improve viability and effectiveness. The bilobed flap is vascularized by the subdermal plexus, making its blood supply random and without a specific pattern, although the size of the flap was quite large. Lymphatic drainage issues are common in the first week after surgery. To prevent these, care should be taken during the procedure, such as gentle handling of tissues, minimizing trauma, and maintaining proper skin tension. After surgery, measures like elevating the extremities, ensuring proper hydration, and monitoring blood pressure to avoid hypotension are crucial. 5

We used a bilobed flap significantly larger than the standard size, measuring about 25 cm. To our knowledge, this is the largest bilobed flap used to date. With this innovation, the patient showed highly satisfactory outcomes, with no observed donor site morbidity, and the bilobed flap remained viable, yielding good clinical results. Compared to the other reports by Mashrah et al., Zwierz et al., and Hasan et al., stated the advantage of the bilobed flap on large defect coverage, good cosmetic results, excellent regional skin color match, and well-padded prosthesis fitting, but without better functional results of joint involvement. 5 9 12

Mashrah et al. reported successful use of bilobed flaps to close donor site defects after radial forearm free flap surgery, although their report only included defects up to 5 cm in size, much smaller than those in our study. The advantages of using a bilobed flap to close donor sites include preventing additional skin grafting scars and achieving uniform skin color with surrounding tissue. 9 However, this technique has some limitations. It requires skilled technique and accurate measurements of angles and tissue size. Incorrect measurements can lead to tension or excess skin after the procedure, also it is essential to ensure the patient's age, nutritional status, and absence of scarring in the flap area and no vascular compromise in the membrane layer to preserve flap viability. Furthermore, the donor site defect from the scapular flap limited alignment with the relaxed skin tension lines. Therefore, we designed the second lobe to match the first in width and height, differing from the conventional design, which enabled tension-free closure and preserved shoulder abduction.

### Conclusion

This case highlights the effectiveness of using a bilobed flap to close large donor site defects after scapular flap harvest. By expanding the size of the bilobed flap beyond traditional limits, this approach demonstrates significant clinical and functional benefits, offering a novel method to protect donor site morbidity of the scapular and parascapular area, and address the challenges associated with large soft tissue reconstruction.
